# Impact of a Nurse-Led Intervention on Depression and Anxiety Among Mothers of Children Admitted to Selected Intensive Care Units: A Quasi-experimental Study

**DOI:** 10.7759/cureus.115059

**Published:** 2026-08-24

**Authors:** Chandravathi KS, Komala HK, Nalini M Nuins

**Affiliations:** 1 Psychiatric Nursing, Adichunchanagiri University, B.G. Nagara, Bellur, IND; 2 Community Health Nursing Department, BGS Institute of Nursing Sciences, Adichunchanagiri University, B.G. Nagara, Bellur, IND; 3 Department of Psychiatric Nursing, Nitte Usha Institute of Nursing Sciences, Nitte (Deemed to be University), Mangalore, IND

**Keywords:** depression and anxiety, family-centered care, intensive care units (icus), mothers of hospitalized children, nurse-led intervention, psychosocial well-being

## Abstract

Objective: This study aimed to assess the psychosocial well-being of mothers whose children were admitted to specific ICUs and to examine the impact of the organized nurse-led psychosocial support program on the mental health of these mothers.

Methodology: The research utilized a one-group quasi-experimental pretest-posttest framework involving 300 mothers of children placed in the chosen ICUs of Dakshina Kannada district, Karnataka, India. The sampling technique utilized was non-probability convenience sampling. The Depression Anxiety Stress Scale-42 (DASS-42) was utilized to assess depression and anxiety. Following the pretest evaluation, mothers received a structured, nurse-led psychosocial support program. The tool was also utilized for assessment after the test. Data analysis was conducted using descriptive and inferential statistics. The paired t-test was employed to compare pretest and post-test scores, Pearson’s correlation assessed the relationship between depression and anxiety scores, and the chi-square test evaluated the association between psychosocial well-being and chosen demographic variables.

Results: A total of 300 mothers of children admitted to selected ICUs participated in the study. Most mothers were aged 26-35 years, accounting for 185 (61.7%) participants. Before receiving the support package, extremely severe depression was observed among 298 (99.3%) mothers, while severe depression was observed among two (0.7%) mothers. Similarly, all 300 (100.0%) mothers demonstrated extremely severe anxiety. During the post-test assessment, normal depression levels were observed among all 300 (100.0%) mothers. With respect to anxiety, 289 (96.3%) mothers demonstrated normal anxiety levels, whereas 11 (3.7%) mothers had mild anxiety. The average depression score significantly declined from 34.36 ± 2.68 to 4.84 ± 1.71 (t=154.04, p<0.001), whereas the average anxiety score significantly dropped from 34.32 ± 2.58 to 4.57 ± 1.72 (t=165.34, p<0.001). Notable positive correlations were found between depression and anxiety scores in both the pretest (r=0.224, p<0.001) and post-test (r=0.175, p=0.002) evaluations. No significant statistical relationship was observed between initial psychosocial well-being and chosen demographic factors (all p>0.05).

Conclusions: The nurse-led structured psychosocial support package was found to be linked to significant reduction in depression and anxiety levels and significant improvements in socio-emotional outcomes for mothers with children admitted to ICUs. The results emphasize the need to integrate structured psychosocial support into routine nursing care to support caregiver well-being and reinforce family-centered care practices.

## Introduction

Hospitalization of children, particularly admission to intensive care units (ICUs), remains a major healthcare challenge worldwide [1]. In India, childhood hospitalization rates are estimated to range from 31.7 to 33 per 1,000 child-years, with acute respiratory infections, gastrointestinal infections, febrile illnesses, and neonatal conditions being the most common causes of admission [1,2]. Despite substantial improvements in pediatric healthcare services, neonatal and childhood illnesses continue to contribute significantly to hospital admissions and healthcare utilization. Although India has achieved considerable reductions in neonatal mortality over the past decade, mortality and morbidity rates remain higher than global averages, necessitating continued efforts to improve pediatric and neonatal care services [3].

Karnataka is one of the leading states in southern India that has made tremendous strides in maternal and child health. The neonatal mortality rate declined from 41 per 1,000 live births in 2009 to 23 per 1,000 live births in 2019 [4]. The state has been continuing to enhance health care delivery systems and boost child health outcomes through various activities under the National Health Mission and Integrated Child Development Services. However, many of these children need to be admitted to intensive care, and this places a huge emotional and psychological burden on the family.

Parents are often overwhelmed by the admission of a child to the ICU, especially mothers who usually care for children. Anxiety, depression, emotional distress and poor psychosocial functioning can result from uncertainty over the prognosis, disruption of family routines, financial concerns, long-term hospitalization, and separation from the child. Some studies have found that parents of children admitted to a critical care unit have a significant psychological burden, which can negatively impact family functioning, family functioning capacity, and family decision-making [3-7].

Nurses play a pivotal role in supporting families during a child’s hospitalization. Owing to their continuous interaction with both patients and caregivers, nurses are uniquely positioned to provide emotional support, counselling, health education, relaxation techniques, and coping strategies aimed at reducing psychological distress [8]. Nurse-led interventions have demonstrated potential benefits in improving emotional well-being, reducing stress, and enhancing coping abilities among caregivers of hospitalized children. However, despite growing recognition of the psychosocial challenges faced by mothers of critically ill children, limited evidence is available regarding the effectiveness of structured nurse-led interventions for improving psychosocial well-being among mothers of children admitted to ICUs in the Indian setting [9].

While there have been some studies on the psychosocial well-being of parents of hospitalized children [10-13], there is limited evidence that structured packages of nurse-led intervention are effective in promoting the psychosocial well-being of parents of children admitted to the ICU in India. In addition, little data exist on the association of psychosocial health with demographic factors among this population. So, the present study was conducted to fill this gap and to assess the effectiveness of the intervention package conducted by nurses among mothers of children admitted to selected ICUs. This study aimed to assess the psychosocial well-being of mothers whose children were admitted to specific ICUs and to examine the impact of the organized nurse-led psychosocial support program on the mental health of these mothers. Furthermore, the study investigated the correlation between depression and anxiety scores and selected demographic parameters of the respondents.

A preliminary study from our research group previously evaluated the effectiveness of a nurse-led intervention among 30 mothers of children admitted to intensive care units. It demonstrated significant improvements in depression, anxiety, and stress following the intervention [14]. However, the limited sample size restricted the generalizability of the findings. Therefore, the present study was undertaken with a substantially larger sample of 300 mothers to provide more robust evidence regarding the effectiveness of the intervention and to perform detailed analyses of demographic characteristics, severity of depression and anxiety, and their associations with selected participant characteristics.

## Materials and methods

Study design and setting

The research utilized a one-group quasi-experimental pretest-posttest framework involving 300 mothers of children admitted to the selected ICUs in Dakshina Kannada district, Karnataka, India. The study was conducted over a period of two years, from April 2024 to April 2026.

Study participants

The study population comprised mothers of children admitted to the selected ICUs during the study period. A total of 300 mothers participated in the study. Non-probability convenience sampling was used for participant recruitment. Mothers who met the eligibility criteria and agreed to participate were included in the study. Written informed consent was obtained from all participants before data collection.

Study instrument

The information was collected using a semi-structured questionnaire consisting of two sections. The initial section included demographic traits such as age, level of education, job, number of kids, family structure, place of living, etc. The second part consisted of an emotion-focused assessment of depression and anxiety, known as the Depression Anxiety Stress Scale-42 (DASS-42) [15], which is a standardized self-assessment survey. The greater the scores, the more mental anguish.

Intervention and data collection procedure

Following the baseline assessment, participants underwent a nurse-led psychosocial intervention consisting of 12 sessions incorporating counseling, meditation, and yoga techniques to improve psychosocial well-being. The intervention was provided as a structured psychosocial support package addressing the emotional and psychological needs of mothers whose children were admitted to the intensive care units. The pretest assessment of depression and anxiety was performed using the DASS-42 before initiation of the intervention. Following completion of the 12-session intervention, a post-test assessment was conducted using the same DASS-42 instrument to evaluate changes in depression and anxiety levels. Data collection was performed personally by the investigators after obtaining permission from the concerned authorities and written informed consent from the participants.

Ethical considerations

All participants in the study provided informed consent, and the confidentiality of the collected data was maintained throughout the research, which received approval from the Institutional Ethics Committee of Adichunchanagiri Institute of Medical Sciences (Reg No: 20PPN001).

Statistical analysis

The data was analyzed using descriptive and inferential statistics. Demographic features and psychosocial well-being scores were compiled using frequency, percentage, mean, standard deviation, and median. To assess the success of the nurse-led intervention, the pretest and post-test scores were compared and analyzed with a paired t-test. The connection between depression and anxiety scores was examined through the Karl Pearson correlation coefficient. At the same time, the relationship between psychosocial well-being and specific demographic factors was assessed using the chi-square test and Fisher’s exact test. A p-value lower than 0.05 was considered significant.

## Results

A total of 185 (61.7%) mothers were aged 26-35 years. Most fathers and mothers were graduates, accounting for 118 (39.3%) and 140 (46.7%), respectively. Nearly half of the families had one child, with 142 (47.3%) reporting a single child. The majority of participants were Hindu (224, 74.7%). Regarding occupation, 106 (33.6%) fathers were employed in the private sector, while 150 (50.0%) mothers were housewives. Most participants resided in rural areas, with 185 (61.7%) living in villages, and 216 (72.0%) families were below the poverty line. A total of 204 (68.0%) participants belonged to nuclear families. The majority of children were first-born (172, 57.3%) and were primarily cared for by their parents (224, 74.7%). More than half of the children were partially dependent for activities of daily living, accounting for 160 (53.3%), and most mothers reported receiving support from their spouse, with 279 (93.0%) responding affirmatively (Table 1).

**Table 1:** Distribution of respondents according demographic profile of the mother (N = 300) Data are presented as number (N) and percentage (%)

Variables		Total count	Percentage %
Age of the mother in years	18-25	44	14.7%
	26-35	185	61.7%
	36-45	71	23.7%
Education of father	Secondary school leaving	44	14.7%
	Pre-university course	43	14.3%
	Graduate	118	39.3%
	Post Graduate	20	6.7%
	Diploma	75	25.0%
Education of mother	Secondary school leaving	33	11.0%
	Pre-university course	43	14.3%
	Graduate	140	46.7%
	Post Graduate	10	3.3%
	Diploma	74	24.7%
Number of children	1 child	142	47.3%
	2 children	116	38.7%
	>2 children	42	14.0%
Religion	Hindu	224	74.7%
	Muslim	43	14.3%
	Christian	11	3.7%
	Others	22	7.3%
Occupation of father	Government	61	21.1%
	Private	106	33.6%
	Business	44	15.2%
	Agriculture	76	26.3%
	Others	11	3.8%
Occupation of mother	Government	21	7.0%
	Private	96	32.0%
	House wife	150	50.0%
	Business	33	11.0%
Area of living	City/Town	115	38.3%
	Village	185	61.7%
Income of father	Above poverty line	84	28.0%
	Below poverty line	216	72%
Income of mother	Above poverty line	84	28.0%
	Below poverty line	216	72%
What is the ordinal position of the child	First child	172	57.3%
	Second child	106	35.3%
	Third child	22	7.3%
Family type	Nuclear family	204	68.0%
	Joint family	96	32.0%
How many siblings does this child have?	None	120	40.0%
	One	136	45.3%
	Two	22	7.3%
	Three	22	7.3%
Who takes care of the child at home?	Parents	224	74.7%
	Grandparents	65	21.7%
	Caretaker	11	3.7%
Is your child dependent for activities of daily living?	Fully	109	36.3%
	Partially	160	53.3%
	Not at all	31	10.3%
Do you get support from your spouse?	Yes	279	93.0%
	No	21	7.0%

The mean pretest depression score was 34.36 ± 2.68, which decreased to 4.84 ± 1.71 in the post-test assessment. Similarly, the mean pretest anxiety score of 34.32 ± 2.58 decreased to 4.57 ± 1.72 following the intervention. The median depression score decreased from 25 in the pretest to 5 in the post-test. By contrast, the median anxiety score reduced from 34 to 4, showing a significant drop both in anxiety and depression scores following the intervention (Table 2).

**Table 2:** Descriptive statistics of depression and anxiety scores before and after intervention Data are presented as mean ± standard deviation (SD) and median

Variable	Minimum	Maximum	Mean ± SD	Median
Pretest depression score	25	40	34.36 ± 2.68	25
Post-test depression score	0	9	4.84 ± 1.71	5
Pretest anxiety score	28	40	34.32 ± 2.58	34
Post-test anxiety score	1	9	4.57 ± 1.72	4

Before the nurse-led intervention, almost all mothers had extremely severe depression, with 298 (99.3%) falling in this category, while two (0.7%) had severe depression. Following the intervention, all mothers, 300 (100.0%), were classified as having normal depression levels. Similarly, all mothers, 300 (100.0%), had extremely severe anxiety during the pretest assessment. Two hundred eighty-nine (96.3%) mothers had normal anxiety levels after the intervention, while 11 (3.7%) mothers had mild anxiety. None of the mothers had moderate, severe, or extremely severe anxiety levels during the post-test assessment. These results suggest that the nurse-led intervention was highly effective in reducing depression and anxiety scores (Table 3).

**Table 3:** Distribution of depression and anxiety severity before and after nurse-led intervention Data are presented as number (N) and percentage (%)

Variables	Pretest n (%)	Post-test n (%)
Depression level		
Normal (0-9)	0 (0%)	300 (100%)
Mild (10-13)	0 (0%)	0 (0%)
Moderate (14-20)	0 (0%)	0 (0%)
Severe (21-27)	2 (0.7%)	0 (0%)
Extremely severe (≥28)	298 (99.3%)	0 (0%)
Anxiety level		
Normal (0-7)	0 (0%)	289 (96.3%)
Mild (8-9)	0 (0%)	11 (3.7%)
Moderate (10-14)	0 (0%)	0 (0%)
Severe (15-19)	0 (0%)	0 (0%)
Extremely severe (≥20)	300 (100%)	0 (0%)

The nurse-led intervention resulted in a substantial reduction in both depression and anxiety scores among mothers. The mean depression score decreased from 34.36 ± 2.68 in the pretest to 4.84 ± 1.71 in the post-test, with a mean reduction of 29.52 points and an 85.91% decrease. Similarly, the mean anxiety score decreased from 34.32 ± 2.58 to 4.57 ± 1.72, with a mean reduction of 29.75 points and an 86.68% decrease. The results of the paired t-test were highly statistically significant, with a decrease in depression scores (t = 154.04, p < 0.001) and a decrease in anxiety scores (t = 165.34, p < 0.001), showing that the nurse-led intervention had a positive effect on reducing depression and anxiety in the mothers of children who were admitted to the ICUs (Table 4).

**Table 4:** Effectiveness of nurse-led intervention on depression and anxiety scores Data are presented as mean ± standard deviation (SD). Statistical analysis was performed using the paired t-test. A p-value <0.05 was considered statistically significant * shows significant p-value

Variable	Pretest Mean ± SD	Post-test Mean ± SD	Mean difference	SD of difference	Percentage reduction (%)	t-value	df	p-value
Depression score	34.36 ± 2.68	4.84 ± 1.71	29.52	3.32	85.91	154.04	299	<0.001*
Anxiety score	34.32 ± 2.58	4.57 ± 1.72	29.75	3.12	86.68	165.34	299	<0.001*

A significant positive correlation was noted between depression scores and anxiety, both prior to and following the nurse-led intervention. In the pretest assessment, there was a weak positive correlation between depression scores and anxiety scores (r = 0.224, p < 0.001). The same was true for the depression and anxiety scores in the post-test assessment (r = 0.175, p = 0.002). The results show that there was a trend for higher depression scores to be correlated with higher anxiety scores in both assessments for mothers (Table 5).

**Table 5:** Correlation between depression and anxiety scores * shows significant p-value Statistical analysis was performed using Pearson’s correlation coefficient (r). A p-value <0.05 was considered statistically significant

Assessment	Variables	Pearson correlation coefficient (r)	p-value
Pretest	Depression score vs anxiety score	0.224	<0.001*
Post-test	Depression score vs anxiety score	0.175	0.002*

The connection between pretest anxiety and specific demographic factors was examined using the Chi-square test. No significant correlation was found between the pretest anxiety level and the subsequent variables: mother's age (p = 0.223); father's education (p = 0.733); mother's education (p = 0.153); number of children (p = 0.614); religion (p = 0.366); father's occupation (p = 0.327); mother's occupation (p = 0.678); living area (p = 0.187); father's income (p = 0.213); mother's income (p = 0.105); child's ordinal position (p = 0.304); family structure (p = 0.560); number of siblings (p = 0.562); child's primary caregiver (p = 0.802); child's dependency for daily living activities (p = 0.440); and spousal support (p = 0.286). The findings from this study indicated that pretest anxiety levels did not correlate with any demographic characteristics of the mothers whose children were hospitalized in ICUs (Table 6).

**Table 6:** Association between pretest anxiety levels and selected demographic characteristics Data are presented as number (N) and percentage (%). Statistical analysis was performed using the chi-square (χ²) test. A p-value <0.05 was considered statistically significant df: degrees of freedom

Variables		Anxiety level (< median score 34)		Anxiety level (≥ median score 34)		Chi-square	df	p-value
		N	%	N	%			
Age of the mother in years	18-25	13	29.5%	31	70.5%	3.004	2	0.223
	26-35	81	43.8%	104	56.2%			
	36-45	30	42.3%	41	57.7%			
Education of father	Secondary school leaving	19	43.2%	25	56.8%	2.076	4	0.733
	Pre-university course	20	46.5%	24	53.3%			
	Graduate	50	42.4%	68	57.6%			
	Post Graduate	9	45%	11	55%			
	Diploma	26	34.7%	49	65.3%			
Education of mother	Secondary school leaving	13	39.4%	20	60.6%	6.711	4	0.153
	Pre-university course	13	30.2%	30	69.8%			
	Graduate	67	47.9%	73	52.1%			
	Post Graduate	2	20%	8	80%			
	Diploma	29	39.2%	45	60.8%			
Number of children	1 child	56	39.4%	86	60.6%	0.976	2	0.614
	2 children	52	44.8%	64	55.2%			
	>2 children	16	38.1%	26	61.9%			
Religion	Hindu	94	42%	130	58%	3.168	3	0.366
	Muslim	20	46.5%	23	53.5%			
	Christian	2	18.2%	9	81.8%			
	Others	8	36.4%	14	63.6%			
Occupation of father	Government	27	44.3%	34	55.7%	4.635	4	0.327
	Private	33	34%	64	66%			
	Business	23	52.3%	21	47.7%			
	Agriculture	31	40.8%	45	59.2%			
	Others	4	36.4%	7	63.6%			
Occupation of mother	Government	11	52.4%	10	47.6%	1.519	3	0.678
	Private	38	39.6%	58	60.4%			
	House wife	60	40%	90	60%			
	Business	15	45.5%	18	54.5%			
Area of living	City/Town	53	46.1%	62	53.9%	1.738	1	0.187
	Village	71	38.4%	114	61.6%			
Income of father	Above poverty line	39	46.4%	45	53.6%	3.093	2	0.213
	Below poverty line	79	38.3%	127	61.7%			
Income of mother	Above poverty line	50	47.6%	55	52.4%	2.632	1	0.105
	Below poverty line	74	37.9%	121	62.1%			
What is the ordinal position of the child	First child	65	37.8%	107	62.2%	2.381	2	0.304
	Second child	50	47.2%	56	52.8%			
	Third child	9	40.9%	13	59.1%			
Family type	Nuclear family	82	40.2%	122	59.8%	0.340	1	0.560
	Joint family	42	3.8%	54	56.3%			
How many siblings does this child have?	None	46	38.3%	74	61.7%	2.049	3	0.562
	One	57	41.9%	79	58.1%			
	Two	9	40.9%	13	59.1%			
	Three	12	54.5%	10	45.5%			
Who takes care of the child at home?	Parents	95	42.4%	129	57.6%	0.440	2	0.802
	Grandparents	25	38.5%	40	61.5%			
	Caretaker	4	36.4%	7	63.6%			
Is your child dependent for activities of daily living?	Fully	48	44%	61	56%	1.644	2	0.440
	Partially	61	38.1%	99	61.9%			
	Not at all	15	48.4%	16	51.6%			
Do you get support from your spouse?	Yes	113	40.5%	166	59.5%	1.137	1	0.286
	No	11	52.4%	10	47.6%			

The chi-square test was employed to examine the relationship between pretest depression levels and chosen demographic traits. There was no significant statistical correlation between pretest depression scores and the age of the children's mothers (p = 0.954), the education level of the fathers (p = 0.800), the education level of the mothers (p = 0.247), the number of children (p = 0.178), the child's religious affiliation (p = 0.334), the occupation of the fathers (p = 0.798), the occupation of the mothers (p = 0.821), the living area (p = 0.434), the income of the fathers (p = 0.538), the income of the mothers (p = 0.520), the child's ordinal position (p = 0.717), the family type (p = 0.713), the number of siblings (p = 0.566), the main caregiver of the child (p = 0.783), the child's dependence on daily living activities (p = 0.604), or spousal assistance (p = 0.740). No notable correlation existed between pretest depression levels and any demographic traits of mothers with children in ICUs (Table 7).

**Table 7:** Association between pretest depression levels and selected demographic characteristics Data are presented as number (N) and percentage (%). Statistical analysis was performed using the chi-square (χ²) test. A p-value <0.05 was considered statistically significant df: degrees of freedom

Variables		Depression level (< median score 35)		Depression level (≥ median score 35)		Chi-square test	df	p-value
		Total number (N)	%	Total number (N)	%			
Age of the mother in years	18-25	20	45.5%	24	54.5%	0.094	2	0.954
	26-35	85	45.9%	100	54.1%			
	36-45	34	47.9%	37	52.1%			
Education of father	Secondary school leaving	21	47.7%	23	52.3%	1.649	4	0.800
	Pre-university course	19	44.2%	24	55.8%			
	Graduate	52	44.1%	66	55.9%			
	Post Graduate	8	40%	12	60%			
	Diploma	39	52%	36	48%			
Education of mother	Secondary school leaving	16	48.5%	17	51.5%	5.413	4	0.247
	Pre-university course	21	48.8%	22	51.2%			
	Graduate	60	42.9%	80	57.1%			
	Post Graduate	8	80%	2	20%			
	Diploma	34	45.9%	40	54.1%			
Number of children	1 child	59	41.5%	83	58.5%	3.457	2	0.178
	2 children	56	48.3%	60	51.7%			
	>2 children	24	57.1%	18	42.9%			
Religion	Hindu	105	46.9%	119	53.1%	3.398	3	0.334
	Muslim	23	53.5%	20	46.5%			
	Christian	3	27.3%	8	72.7%			
	Others	8	36.4%	14	63.6			
Occupation of father	Government	31	50.8%	30	49.2%	1.660	4	0.798
	Private	44	45.4%	53	54.6%			
	Business	18	40.9%	26	59.1%			
	Agriculture	37	48.7%	39	51.3%			
	Others	4	36.4%	7	63.6%			
Occupation of mother	Government	9	42.9%	12	57.1%	0.920	3	0.821
	Private	45	46.9%	51	53.1%			
	House wife	72	48%	78	52%			
	Business	13	39.4%	20	60.6%			
Area of living	City/Town	50	43.5%	65	56.5%	0.611	1	0.434
	Village	88	48.1%	96	51.9%			
Income of father	Above poverty line	38	45.2%	46	54.8%	1.241	2	0.538
	Below poverty line	98	47.6%	108	52.4%			
Income of mother	Above poverty line	46	43.8%	59	56.2%	0.414	1	0.520
	Below poverty line	93	47.7%	102	52.3%			
What is the ordinal position of the child	First child	78	45.3%	94	54.7%	0.664	2	0.717
	Second child	49	46.2%	57	53.8%			
	Third child	12	54.5%	10	45.5%			
Family type	Nuclear family	96	47.1%	108	52.9%	0.135	1	0.713
	Joint family	43	44.8%	53	55.2%			
How many siblings does this child have?	None	50	41.7%	70	51.5%	2.030	3	0.566
	One	66	48.5%	70	51.5%			
	Two	12	54.5%	10	45.5%			
	Three	11	50%	11	50%			
Who takes care of the child at home?	Parents	104	46.4%	120	53.6%	0.489	2	0.783
	Grandparents	31	47.7%	34	52.3%			
	Caretaker	4	36.4%	7	63.6%			
Is your child dependent for activities of daily living?	Fully	53	48.6%	56	51.4%	1.007	2	0.604
	Partially	70	43.8%	90	56.3%			
	Not at all	16	51.6%	15	48.4%			
Do you get support from your spouse?	Yes	130	46.6%	149	53.4%	0.110	1	0.740
	No	9	42.9%	12	57.1%			

## Discussion

The current investigation expands upon our previously published pilot study involving 30 mothers [14]. Although the earlier study established the beneficial effects of a nurse-led intervention on depression, anxiety, and stress, the present research enrolled a substantially larger sample of 300 mothers, enabling a more comprehensive evaluation through detailed demographic profiling, severity classification, correlation analyses, and examination of the relationships between psychosocial outcomes and participant characteristics.

This study evaluated the effectiveness of a structured nurse-led intervention in improving the psychosocial well-being of mothers whose children were admitted to selected ICUs. Extremely high levels of depression and anxiety were observed before the intervention, followed by substantial reductions after implementation of the psychosocial support package. These findings highlight the considerable emotional burden experienced by mothers of critically ill children and emphasize the important contribution of structured nursing interventions in reducing psychological distress.

A key finding was the exceptionally high prevalence of psychological distress before the intervention. Nearly all participants (99.3%) demonstrated extremely severe depression, while every mother (100%) reported extremely severe anxiety. Similar observations have been reported by Stremler et al. [12], who found that parents of critically ill hospitalized children commonly experience significant anxiety, depression, and emotional distress. Manning et al. [13] further described how uncertainty, fear, helplessness, and disruption of family roles contribute substantially to parental psychological stress during ICU admission. Carter et al. [16] likewise documented high levels of anxiety and depression among parents of critically ill children, identifying the ICU environment itself as a major source of emotional strain. In addition, Colville and Pierce [17] demonstrated that many parents develop symptoms resembling post-traumatic stress following their child's critical illness and hospitalization. Collectively, these studies reinforce that admission of a child to an ICU represents a profound psychological stressor for mothers.

Following the implementation of the nurse-led intervention, depression scores declined markedly. The mean depression score decreased from 34.36 ± 2.68 at baseline to 4.84 ± 1.71 after the intervention, representing an 85.91% reduction, and all participants were classified within the normal range during the post-test assessment. These findings are consistent with those of Melnyk et al. [18], who demonstrated that structured psychosocial support effectively reduced depressive symptoms among parents of critically ill children. The Creating Opportunities for Parent Empowerment (COPE) program developed by Melnyk and colleagues improved parental coping, alleviated psychological distress, and promoted family adaptation during hospitalization. Comparable improvements were also reported by Davila and Segre [19], who observed significant reductions in depressive symptoms among mothers of hospitalized infants following nurse-led psychosocial interventions. Similarly, Shaw et al. [20] found that structured family-centered interventions enhanced parents' emotional well-being while reducing psychological morbidity. The marked improvement observed in the present study may be associated with the comprehensive intervention package, which integrated counseling, emotional support, health education, and coping guidance. However, given the absence of a control group, other factors that may have contributed to the observed changes cannot be excluded.

Anxiety levels also showed substantial improvement following the intervention. The mean anxiety score declined from 34.32 ± 2.58 before the intervention to 4.57 ± 1.72 afterward, corresponding to an 86.68% reduction. Moreover, 96.3% of mothers demonstrated normal anxiety levels during the post-test assessment. These findings agree with those of Kasparian et al. [21], who reported that psychosocial support programs significantly reduced anxiety among parents of children requiring intensive medical care. Melnyk et al. [18] similarly observed significantly lower anxiety levels among parents receiving structured psychological support compared with those receiving routine care alone. McCarthy et al. [22] further demonstrated that counseling-based interventions enhanced parental coping and significantly reduced anxiety during childhood hospitalization. Together, these findings indicate that structured nurse-led psychosocial interventions are highly effective in alleviating the emotional distress associated with a child's critical illness.

Meditation and yoga formed important components of the intervention and may have contributed to the observed improvements in psychosocial well-being. Khalsa and Butzer [23] reported that yoga-based interventions significantly reduced symptoms of anxiety, depression, and perceived stress among caregivers. Likewise, Pascoe and Bauer [24] demonstrated that mindfulness-based meditation enhanced emotional regulation while reducing stress-related symptoms among individuals with caregiving responsibilities. By promoting relaxation, strengthening coping skills, and attenuating physiological stress responses, these practices may partly explain the substantial improvements observed in the present study.

A statistically significant positive correlation was identified between depression and anxiety scores in both the pretest and post-test assessments, indicating that mothers with higher depression scores also tended to report greater anxiety. Similar findings were reported by Stremler et al. [12], who identified a strong association between depressive and anxiety symptoms among parents of critically ill children. Pinquart and Shen [25] likewise concluded in their meta-analysis that depression and anxiety frequently coexist among caregivers because they share common risk factors, including caregiver burden, emotional exhaustion, uncertainty, and concerns regarding patient outcomes. Comparable findings were also reported by Pochard et al. [26], who documented concurrent anxiety and depression among relatives of ICU patients. These findings suggest that improvement in one psychological domain is likely to be accompanied by improvement in the other.

No statistically significant associations were identified between baseline depression or anxiety levels and any of the selected demographic variables, including age, education, occupation, family type, household income, area of residence, number of children, spousal support, or the child's dependency status. Balluffi et al. [27] likewise reported that parental psychological distress was influenced primarily by illness severity and the hospitalization experience rather than demographic factors. Board and Ryan-Wenger [28] further concluded that emotional responses to pediatric critical illness are largely universal across diverse socioeconomic and educational backgrounds. These findings support the routine provision of psychosocial support to all mothers of children admitted to ICUs, irrespective of their demographic characteristics.

The considerable effectiveness of the nurse-led intervention further underscores the pivotal role of nurses in promoting caregivers’ psychosocial well-being and strengthening family-centered care. Through continuous interaction with patients and families, nurses are uniquely positioned to provide emotional support, counseling, health education, and practical coping strategies. Addressing the psychological needs of mothers is an important component of family-centered care, as caregiver well-being may influence family adaptation and participation in the child’s care. Incorporating structured psychosocial interventions into routine ICU nursing practice may therefore strengthen family-centered care, facilitate caregiver adaptation, and reduce the psychological burden associated with childhood critical illness, ultimately supporting the well-being of both caregivers and their children.

Limitations

Several limitations should be considered when interpreting these findings. First, the one-group pretest/post-test design without a control group limits the ability to establish a definitive causal relationship between the intervention and the observed improvements. Second, the use of non-probability convenience sampling may restrict the generalizability of the findings to other populations and healthcare settings. Third, psychosocial well-being was assessed using self-reported questionnaires, making the results susceptible to recall, response, and social desirability biases. Fourth, outcomes were evaluated immediately after completion of the intervention, precluding assessment of its long-term effectiveness. Fifth, the study included only mothers; therefore, the findings may not be generalizable to other caregivers, such as fathers or grandparents, who may respond differently to a child's hospitalization. Finally, potentially important clinical and psychosocial factors, including illness severity, duration of ICU stay, previous mental health status, and availability of social support, were not examined and may have influenced the observed outcomes.

Despite these limitations, the findings provide strong evidence supporting the effectiveness of structured nurse-led psychosocial interventions in improving the mental well-being of mothers of critically ill children. The results reinforce the importance of integrating organized psychosocial support into routine nursing practice as a fundamental component of family-centered pediatric intensive care.

## Conclusions

The findings demonstrate a substantial reduction in depression and anxiety scores among mothers following the nurse-led psychosocial intervention. The results suggest that structured nurse-led psychosocial support incorporating counseling, meditation, and yoga may be beneficial in addressing psychological distress among mothers of children admitted to ICUs. However, because this study employed a one-group quasi-experimental pretest-posttest design without a control group, the observed improvements cannot be attributed solely to the intervention, and causal conclusions should therefore be interpreted cautiously. Future studies using controlled or randomized designs with larger and more diverse samples are recommended to further evaluate the effectiveness and causal impact of nurse-led psychosocial interventions in this population.
